# Patellofemoral arthroplasty provides similar long‐term survival rate and complications with better clinical outcomes compared to facetectomy for the treatment of isolated patellofemoral osteoarthritis

**DOI:** 10.1002/jeo2.70136

**Published:** 2025-01-10

**Authors:** Jan Martinez‐Lozano, Lucas Martorell‐de Fortuny, Lidia Ana Martin‐Domínguez, Raul Torres‐Claramunt, Juan Sánchez‐Soler, Simone Perelli, Pedro Hinarejos, Joan Carles Monllau

**Affiliations:** ^1^ Orthopaedic Department, Hospital del Mar Universitat Autònoma Barcelona Barcelona Spain; ^2^ ICATKnee, Institut Català de Traumatologia i Medicina de l'Esport (ICATME), Hospital Universitari Dexeus Universitat Autònoma Barcelona Barcelona Spain; ^3^ Knee Surgery Department, Barcelona Trauma Institute Centro Médico Teknon Barcelona Spain

**Keywords:** isolated patellofemoral osteoarthritis, lateral facetectomy, patellofemoral arthroplasty

## Abstract

**Purpose:**

This study aimed to analyse the clinical outcomes and survival of patellofemoral arthroplasty (PFA) in treating isolated patellofemoral osteoarthritis (IPFOA) at our centre. The secondary objective was to compare these results with a historical cohort treated with partial lateral facetectomy plus Insall realignment (PLFIR). We hypothesised that clinical outcomes and survival with PFA are superior to PLFIR and comparable to the literature.

**Methods:**

A retrospective analysis of 120 patients with IPFOA was conducted. The PFA series included 33 patients treated between 2012 and 2019 with a minimum follow‐up of 5 years (range 1.2–12.1 years). The PLFIR historical cohort treated between 1995 and 2002 (range 4.1–15.7 years) consisted of 87 patients. Preoperative and post‐operative clinical outcomes were assessed using the Knee Society Score (KSS) and Kujala score, and survivorship was evaluated via Kaplan–Meier analysis. Cox regression analysis was used to identify factors influencing surgical failure.

**Results:**

The PFA group demonstrated a 75.8% survival rate at 10 years, with a 24.2% failure rate requiring conversion to total knee arthroplasty (TKA). In the PLFIR group, the 10‐year survival rate was 79.3%, although 26.4% required TKA. Both groups exhibited significant improvements in KSS and Kujala score, with PFA showing superior Kujala score improvement (*p* = 0.012). No statistically significant difference in survival between the two groups was observed at 10 years (*p* = 0.056), but PFA showed better long‐term clinical outcomes.

**Conclusions:**

PFA demonstrated comparable survival rates to PLFIR in the treatment of IPFOA. Despite a higher initial failure rate, PFA showed a potential for greater improvement in the long term, particularly in terms of anterior knee pain.

**Level of Evidence:**

Level IV, retrospective case series analysis compared with a historical cohort.

**Abbreviations**
BMI: body mass index
CT: computed tomography
C‐D: Caton–Deschamps
IPOA: isolated patellofemoral osteoarthritis
KL: Kellgren–Lawrence
KSS: Knee Society Score
MTFA: mechanical tibiofemoral angle
OR: odds ratio
PFA: patellofemoral arthroplasty
PFOA: patellofemoral osteoarthritis
PLFIR: partial lateral facetectomy plus Insall realignment
PTA: patellar tilt angle
SD: standard deviation
TKA: total knee arthroplasty
TT‐TG: tibial tubercle‐trochlear groove

## INTRODUCTION

Patellofemoral osteoarthritis (PFOA) usually occurs with involvement of the femorotibial compartments, but in 8.3%–11.7% of the general population occurs as isolated patellofemoral osteoarthritis (IPFOA) [6, 13, 18]. In 28%–42% of cases, the aetiology is influenced by alterations in joint biomechanics [11, 24]. For this reason, most surgical techniques aimed at improving symptoms have focused on correcting these biomechanical alterations, especially patellar maltracking, through realignment of the extensor apparatus.

Although anterior tibial tuberosity osteotomies, such as Maquet+ [16] or Fulkerson procedures [9], have been effective in some cases, they have not provided a solution for most cases of IPFOA, likely because they leave an already established arthropathy untreated [2]. In the 1990s, Iwano et al. noted that 89% of IPFOA cases occurred in the lateral facet [12], leading to the popularity of partial lateral facetectomy [17]. In subsequent years, multiple studies were published treating IPFOA with lateral facetectomy combined with realignment techniques [3, 4, 15, 19, 20, 30]. Despite the good results reported, these techniques have been largely relegated in recent years, with the last publication dating back to 2014 [19].

The reason for this decline was the advent of patellofemoral arthroplasty (PFA). The first‐generation inlay prostheses, implanted from the 1970s onwards, had poor outcomes, with revision and dissatisfaction rates of around 20% and 40%, respectively [1]. This led to the development of new onlay models in the 1990s, which soon became known as second‐generation PFAs. These implants improved outcomes by halving revision and dissatisfaction rates [22, 26]. Although the ideal treatment of IPFOA remains a controversial topic, the current trend is the increasing use of PFAs. According to data from the Australian Orthopaedic Association National Joint Replacement Registry, PFA placement has increased by 45% in the last 5 years [10].

In our centre, partial lateral facetectomy plus Insall realignment (PLFIR) was traditionally performed, and despite the good results, the indication was changed to PFA following the global trend. For this reason, the main objective of our study is to analyse the clinical results and survival of PFA in treating IPFOA in our centre. The secondary objective is to compare the outcomes of both techniques. Our hypothesis is that the clinical outcomes and survival with PFA are good, better than those obtained in the past with PLFIR, and comparable to those reported in the literature.

## METHODS

This is a retrospective analysis of a case series of IPFOA treated by PFA at a single centre, with subsequent comparison to a historical IPFOA cohort treated by PLFIR at the same centre. The study received the institutional review board's approval of our Ethical Committee (2023/14‐TRA‐DEX), signed on 1 August 2014 (acta no. 16/2024). The historical IPFOA cohort treated with PLFIR from 1995 to 2002 with a minimum follow‐up of 10 years, from which two articles had already been published in 2013 and 2014 [19, 20], was retrieved from our centre's database. For the PFA series, all patients who underwent PFA (Gender Solutions, Zimmer) in our centre until February 2019 were assessed for eligibility criteria. In this period, no patients were treated with lateral facetectomy. The exclusion criteria were: implants other than the Gender Solutions Patellofemoral Joint System, follow‐up less than 5 years, a previous history of patellar fracture or dislocation, and the absence of preoperative clinical tests and full radiological studies to analyse different patellofemoral radiological parameters. These criteria (except from the implant model) were also considered for the PLFIR cohort.

**Figure 1:**
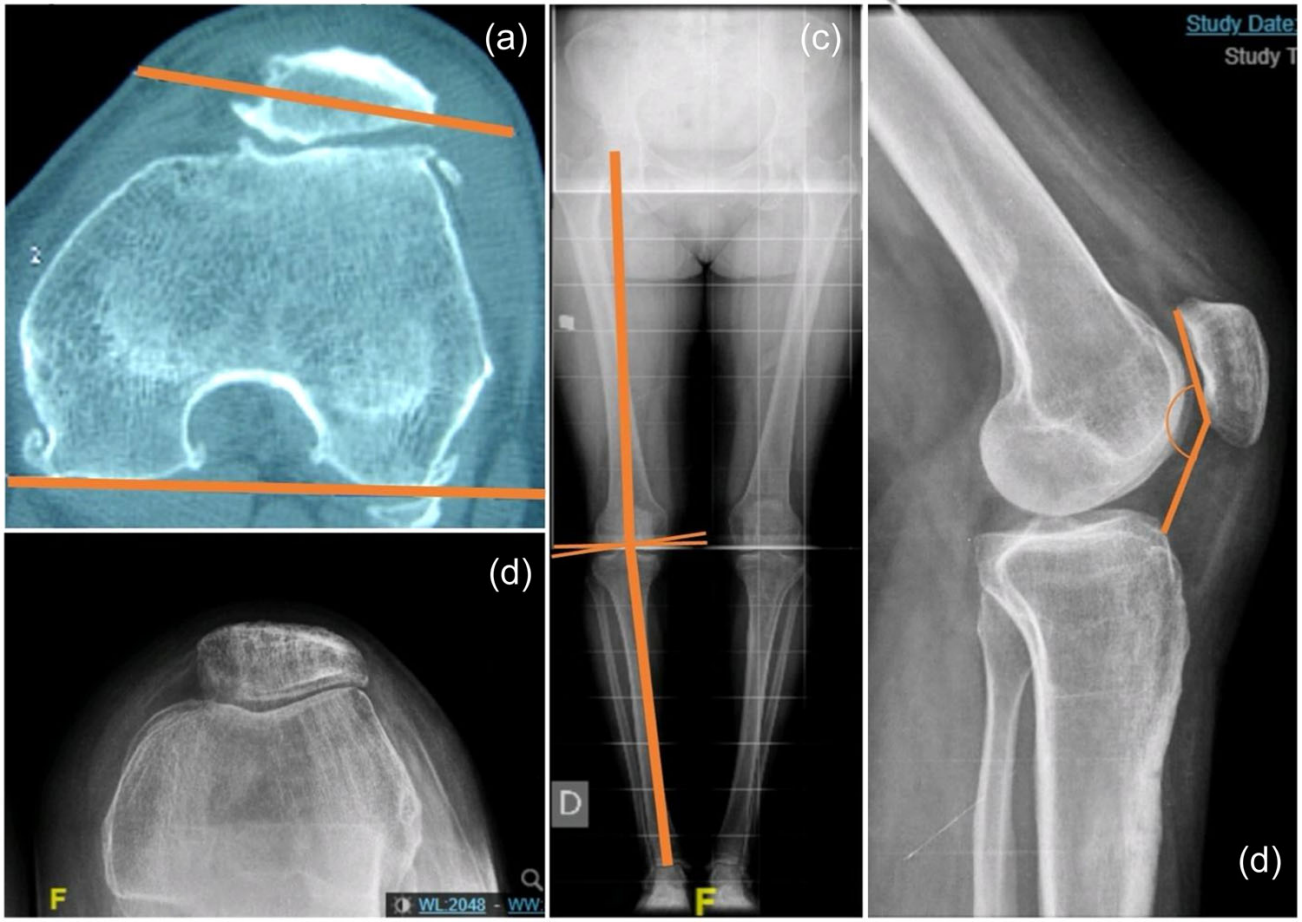
Radiological measurements. The four radiological projections used in the measurements: (a) axial view of the CT scan of the knee. Outlined in orange is the measurement of the patellar tilt angle. The tibial tubercle‐trochlear groove was also obtained. (b) Skyline view to assess patellar morphology and degree of patellofemoral osteoarthritis. (c) Full‐length lower limb x‐ray to assess mechanical femorotibial angle as outlined in orange. (d) Caton–Deschamps index in the lateral 30° view. Also, Dejour index is assessed in this projection. CT, computed tomography.

The variables considered were the same in both the PFA series and the historical cohort. The demographic variables were age, sex, body mass index (BMI) and laterality, which were collected from clinical history. As for the radiological parameters, all were measured by the same senior knee surgeon from the preoperative radiology (computed tomography [CT] and the following radiographic views: full‐length lower extremities, Rosenberg view, true lateral and 30° flexion skyline). Mechanical femorotibial angle (MTFA) was assessed using the full‐length lower extremities view. Patellar height was measured using the Caton–Deschamps (C‐D) index in the lateral view. Patellar morphology was determined with the skyline view according to Wiberg's classification, while patellar tilt angle (PTA) was analysed by CT. The tibial tubercle‐trochlear groove (TT‐TG) distance was also measured by CT. The degree of patellofemoral (PF) and tibiofemoral osteoarthritis was determined using the skyline view according to Iwano's classification and the Rosenberg view according to the Kellgren–Lawrence (KL) classification, respectively. Furthermore, trochlear dysplasia was assessed using Dejour classification [7] (Figure 1). The surgery duration, component size, and the need for additional surgical gestures other than prosthesis implantation were collected from the operative sheet. To assess clinical results, the Knee Society Score (KSS) and Kujala score were used. All patients from the PFA series were scheduled for follow‐up in March 2024 to obtain test results, while clinical outcomes for PLFIR were taken from the database. Finally, all complications during follow‐up were reviewed.

The need for total knee arthroplasty (TKA) conversion was considered the primary end point for the survival analysis. All patients lost during follow‐up due to death, loss of follow‐up or inability to collect post‐operative clinical data were included in the survivorship analysis as censored patients.

### Surgical techniques and post‐operative care

Both PFA and PLFIR procedures were performed by a team of experienced senior knee surgeons. Patients were placed in the supine position with a tourniquet applied at the thigh level. For the PFA procedure, a medial parapatellar approach to the PF joint was used, with a central skin incision. With the patella everted, the trochlea was prepared by functional alignment, adjusting rotation, varus‐valgus tilt and trochlear offset. Patellar denervation and osteophyte exeresis were performed in all cases.

Subsequently, after measurement of the patellar thickness, the patellar cut was performed preserving between 14 and 16 mm of bone depending on the implant size, to prevent overstuffing. The position of the patellar button was adjusted according to the TT‐GT and CD values and according to the tracking achieved after implantation of the test trochlear component. The definitive components were cemented using Gentamicin‐loaded cement (Copal® G, Heraeus or Simplex® G, Stryker). After prosthesis implantation, PF tracking was evaluated. Based on tracking and preoperative study results, additional surgical realignment gestures were performed as necessary.

**Figure 2:**
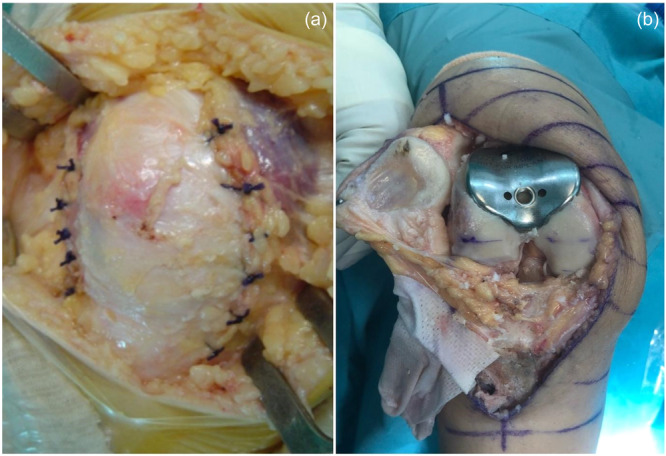
Intraoperative images of the two procedures. In (a), an anterior projection of the lateral facetectomy after the Iwano procedure, while in (b), the femoral tray of the patellofemoral arthroplasty in place with the degenerated articular surface exposed before the implant of the patellar button.

For the PLFIR procedure, a lateral arthrotomy was performed through a central skin incision. The lateral and anterior soft tissues were dissected to expose and excise most of the lateral facet using an oscillating saw. After closing the arthrotomy without tightening the lateral soft tissue structures, proximal soft tissue realignment was performed using the Insall's procedure. Care was always taken to avoid hypercorrection of patellar tilt and shift and to ensure adequate patellar tracking (Figure 2).

All patients underwent the same rehabilitation protocol, starting with continuous passive motion and isometric exercises focused on the quadriceps and gluteal muscles, with weight‐bearing beginning the first day after surgery. Hospital admission lasted until pain was controlled and the patient could independently perform basic daily living activities. After discharge, patients continued rehabilitation at the same hospital three times a week for one month. Once rehabilitation was completed, patients were seen by their surgeon at 1, 3 and 6 months, then 1 year post‐operatively, and annually thereafter.

### Statistical analysis

Descriptive statistics were used to summarise all demographic, surgical, radiographic and functional variables. Qualitative values were expressed as percentages, while mean and standard deviation were used for quantitative variables. A paired two‐tailed sample Student's *t*‐test was used to compare preoperative and post‐operative values, as well as variables between the PFA and facetectomy groups when they followed a normal distribution as tested using the Shapiro–Wilk test. Care must be taken in the association between dysplasia and risk for failure of PFA where the odds ratio (OR) was used to express association altogether with a Fisher exact test to assess for significance of the association. Non‐parametric variables were analysed using the Mann–Whitney *U* test. A *p* < 0.05 was considered the threshold of statistical significance. Survivorship was displayed using a Kaplan–Meier curve. Univariate Cox regression analysis was used to identify factors that could influence the failure of the surgical procedure. All statistical analyses were conducted using SPSS software.

## RESULTS

A total of 120 patients were included, of which 33 were part of the PFA series and 87 of the PLFIR historical cohort. 36 PFAs were implanted in our centre between 2012 and 2019, with 33 patients meeting the eligibility criteria. Two patients had bilateral PFAs. Two out of the three cases excluded had a previous traumatic history of patellar fracture, and the other lacked preoperative clinical tests. One case was lost, and two patients died from unrelated medical causes at 65 and 140 months post‐operatively without requiring a PFA revision.

Although the main indication for PLFIR may be not exactly the same for PFA. Being the lateral facet arthropathy associated with an increased patellar tilt is the main indication for PLFIR, while whether the lateral or medial facets are affected and the PTA are not so important for PFA. Despite this, on reviewing the case series and given that IPFOA only starts in the medial facet in very specific cases associated with a history of dislocation and significant dysplasia, no relevant differences between both treatment groups have been found related to surgical indication.

The mean follow‐up of the PFA series was 6.5 (standard deviation [SD] 3.1, range 1.2–12.1) years. The rest of the demographic, radiological, and intraoperative characteristics are displayed in Tables 1, 2, 3, in comparison with those of the PLFIR historical cohort, which consisted of 87 patients with a mean follow‐up of 9.2 (SD 3.2) years. The only significant differences between the PFA and PLFIR groups were in the PTA, C‐D index and TA‐GT (Table 3). Seven patients of the PFA group presented trochlear dysplasia, while in the PLFIR group, the rate was 14.9%, with no significant differences.

**Table 1:** Comparison of demographic variables between the two groups.

	PFA (33)	PLFIR (87)	*p*
Age (years)	59.33 (SD 10.0)	59.7 (SD 8.1)	n.s.
Weight (kg)	78.91 (SD 14.4)	74.31 (SD 12)	n.s.
Height (cm)	163 (SD 9.4)	162.4 (SD 11.71)	n.s.
Body mass index	29.74 (SD 5.1)	28.2 (SD 6.39)	n.s.
Gender (male)	8 (24.2%)	6 (6.9%)	n.s.
Side (right)	20 (60.6%)	43 (49.4%)	n.s.

Abbreviations: n.s., not statistically significant; PFA, patellofemoral arthroplasty; PFLIR, partial lateral facetectomy plus Insall realignment.

**Table 2:** Intraoperative variables of the patellofemoral arthroplasty group.

Surgical characteristics	
Associated procedures	5 (15.1%)
Fulkerson osteotomy	3 (60%)
Lateral retinaculum release	2 (40%)
Patellar lengthening	1 (20%)
Trochlea implant size	
1	3 (9.1%)
2	11 (33.3%)
3	9 (27.3%)
4	7 (21.2%)
5	3 (9.1%)
Patella implant size	
29	5 (15.2%)
32	27 (81.8%)
38	1 (3.0%)

**Table 3:** Comparison of the radiological variables between the two groups.

	PFA (*n* = 33)	PLFIR (*n* = 87)	*p*
PTA	9.39 (SD 5.9)	14.7 (SD 7.3)	0.047
TT‐GT	11.39 (SD 4.6)	8.5 (SD 5.1)	0.005
C‐D index	1.0 (SD 0.1)	0.9 (SD 0.2)	0.01
MTFA			n.s.
Iwano			n.s.
1	1 (3.03%)	0	
2	4 (12.12%)	5 (5.75%)	
3	18 (54.54%)	49 (56.32%)	
4	10 (30.30%)	33 (37.93%)	
Wiberg			N.A
1			
2	11 (33.3%)	N.A.	
3	13 (39.4%)	N.A.	
Dejour			N.A.
Normal	10 (30.3%)	N.A.	
A	12 (36.3%)	N.A.	
B	8 (24.2%)	N.A.	
C	2 (6.1%)	N.A.	
D	1 (3.1%)	N.A.	

Abbreviations: C‐D, Caton–Deschamps; MTFA, mechanical tibiofemoral angle; PFA, patellofemoral arthroplasty; PFLIR, partial lateral facetectomy plus Insall realignment; PTA, patellar tilt angle; N.A., not available; n.s., not statistically significant; TT‐GT, tibial tubercle‐trochlear groove distance; SD, standard deviation.

For PFA, 18.2% (5) of cases required additional surgical procedures. Three patients underwent an anterior tibial tubercle osteotomy, two required a release of the lateral retinaculum (one of them in conjunction with a Fulkerson osteotomy), and one required patellar lengthening. There were no intraoperative complications, but one case of an immediate post‐operative prepatellar tension haematoma that required debridement. Regarding non‐immediate post‐operative complications, there was one case of a periprosthetic distal femoral fracture after a high‐energy accident one year post‐operatively, which was treated with nailing. Both cases progressed satisfactorily after treatment of the complications. In the PLFIR group, there were no intraoperative complications, but there were nine (10.3%) cases of post‐operative complications. Four patients experienced mild complex regional pain syndrome, three had superficial infections, one developed an internal saphenous neuroma, and one had a transient extension lag.

**Figure 3:**
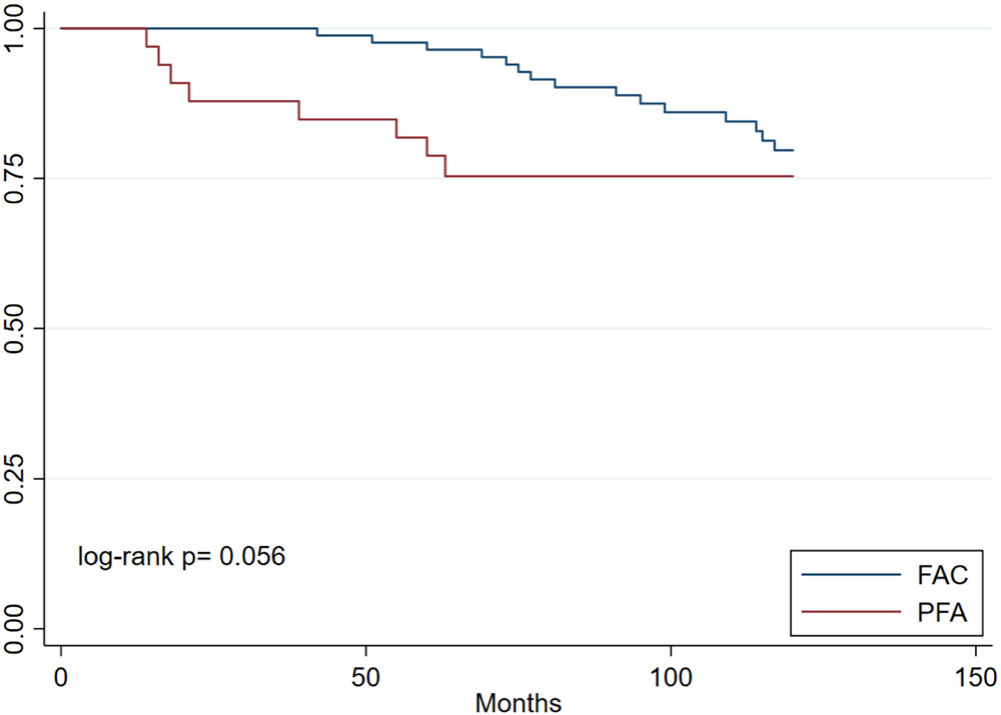
Kaplan–Meier curve showing survivorship at 10 years of the two procedures. The survivorship of the patellofemoral arthroplasty (PFA, red) and Partial lateral facetectomy plus Insall realignment (FAC, blue) are presented in the Kaplan–Meier curve. Difference between the two groups is measured using log‐rank. The value for statistical significance is *p* < 0.005.

The survival analysis of the two cohorts is depicted with the Kaplan–Meier curve in Figure 3. At 10 years of follow‐up, there was no significant difference between the two techniques. In the PFA series, at 10 years, the cumulative survivorship was 75.8%, with the last failure at 5 years of follow‐up. The mean (SD) survival time was 6.5 (3.11) years, with a range of 14–142 months. In the PLFIR historical cohort, the cumulative survival rate at 10 years was 79.3% (*p* = 0.058).

In the shorter term, average survival at 5 years was 93.1% for the PLFIR group while PFA showed survival rates of 78.7% (*p* = 0.001).

In the PFA group, there were eight (24.2%) failures, with four (50%) occurring within the first 18 months after surgery and two (20%) after four and a half years of follow‐up. Except for the cases that required conversion to TKA and the two cases with complications, only one patient underwent additional surgery. This was an arthroscopy for debridement and microfracture of a chondral lesion in the medial compartment, which ultimately led to TKA conversion. In the historical cohort, no surgical procedures other than TKA were performed after the lateral facetectomy plus Insall's procedure. Even the cases with complications did not require further surgery. Only two of the patients that required TKA presented preoperatively a trochlear dysplasia with an OR of 1.2 (SD: 0.2–8.3, no significance). In the PLFIR cohort, 23 out of 87 cases (26.4%) required conversion to TKA.

In terms of clinical outcomes, both techniques demonstrated statistically significant improvement at the end of follow‐up compared to baseline. All data are presented in Table 4. When comparing KSS at the end of follow‐up between the two techniques, there was no significant difference between the groups. Regarding the Kujala score, patients in the PFA group had lower baseline scores compared to those who underwent PLFIR. The final improvement was also greater in the PFA series (*p* = 0.012).

**Table 4:** Comparison of the clinical variables between the two groups.

	PFA (*n* = 33)	PLFIR (*n* = 87)	*p*
KSS functional preoperative	61.47 (SD 10.3)	66.4 (SD 19.8)	n.s.
KSS functional post‐operative	76.59 (SD 18.3)	78.9 (SD 22.1)	n.s.
KSS knee preoperative	65.39 (SD 9.6)	62.3 (SD 13.1)	n.s.
KSS knee post‐operative	85.14 (SD 13.0)	79.8 (SD 14.3)	n.s.
Kujala score preoperative	55.38 (SD 7.3)	60.4 (SD 10.3)	0.020
Kujala score post‐operative	81.14 (SD 16.7)	69.5 (SD 17.5)	0.012

Abbreviations: KSS, Knee Society Score; n.s., not statistically significant; PFA, patellofemoral arthroplasty; PFLIR, partial lateral facetectomy plus Insall realignment; SD, standard deviation.

The univariate Cox analysis of the risk factors for failure is presented in Table 5. Of all the factors studied, only the preoperative MTFA showed a borderline *p*‐value of significance (*p* = 0.085): patients requiring conversion to TKA had a higher varus axis (177°) compared to the other patients (181°). Additionally, patients who required revision were older and required a smaller size of trochlear prosthesis (three out of eight revisions required size 1, while none of the other 25 patients used this size).

**Table 5:** Univariate analysis of risk factors for failure of the patellofemoral arthroplasty (PFA).

	No TKA conversion (*n* = 25)	TKA conversion (*n* = 8)	*p*
Age (years)	57.80 (SD 9.92)	64.13 (SD 9.17)	n.s.
BMI	29.53 (SD 5.19)	30.41 (SD 5.22)	n.s.
KSS functional preoperative	64.60 (SD 9.39)	69.33 (SD 12.10)	n.s.
KSS knee preoperative	61.79 (SD 10.12)	60.00 (SD 13.23)	n.s.
PTA	10.00 (SD 6.02)	7.50 (SD 5.61)	n.s.
TT‐GT	11.44 (SD 4.86)	11.25 (SD 4.17)	n.s.
C‐D Index	1.02 (SD 0.13)	0.96 (SD 0.17)	n.s.
MTFA	181.52 (SD 5.64)	177.75 (SD 3.37)	n.s.

*Note*: The failure of the arthroplasty is considered as a requirement for TKA conversion. Although differences among the two subgroups are present, none of them presents statistical significance.

Abbreviations: BMI, body mass index; C‐D, Caton–Deschamps; KSS, Knee Society Score; MTFA, mechanical tibiofemoral angle; n.s., not statistically significant; PFLIR, partial lateral facetectomy plus Insall realignment; PTA, patellotibial angle; SD, standard deviation; TKA, total knee arthroplasty; TT‐GT, tibial tubercle‐trochlear groove distance.

## DISCUSSION

The main finding of this study is that PFA (Gender Solutions, Zimmer) achieves good clinical results and a 75.8% survivorship with a mean follow‐up of 6.5 years. When comparing these results with those of PLFIR [19, 20], the TKA conversion rate of both techniques is similar, but the clinical outcomes showed lower average values for the PFA group, although without statistical significance (with exception of Kujala). Therefore, it can be stated that our hypothesis has been at least partially confirmed.

To our knowledge, this is the first study comparing the results of PFA with another joint‐preserving technique, as well as the only case series using this prosthesis model with a minimum follow‐up of 5 years. Clinical results are consistent and favourable in the most recent systematic reviews. Focusing solely on onlay PFA results, Villa et al. [29] reported KSS knee and functional scores of 79.7 and 79.3, respectively, at the end of follow‐up, although preoperative values were not provided. Familiari et al. also published good functional outcomes measured by KSS when comparing onlay versus inlay PFA, with an average improvement of 27.6 points in the onlay group, without distinguishing between KSS knee and function [8]. Sava et al., in a comparison of onlay versus inlay designs, reported average improvements of 25 and 30 points in KSS and Kujala score, respectively, and a 4.5‐point improvement on the visual analogue scale when using onlay designs [25].

In our onlay PFA series, KSS results were comparable to those previously referenced. We also used the Kujala score, as Sava et al. did, which has been shown to be superior for detecting anterior knee pain [5]. The mean Kujala score improvement in the PFA series was 25.8 points, which is more than double the minimum detectable change threshold considered by Kunze et al. [14].

When reviewing TKA conversion rates in literature, this consistency diminishes. Vila et al. found a 7.8% TKA conversion rate. Notably, they also reported a 1.4% revision rate without conversion to TKA, but it is surprising that no further surgeries were noted [29]. Familiari et al. also reported a very low 7% rate of TKA conversion but claimed that up to 14.2% of patients required further surgeries other than TKA conversion [8]. On the other hand, Sava et al. published a 21% TKA conversion rate [25] with onlay designs in the same year as Familiari et al. In the Australian registry, the 2019 conversion and revision rates were 17.5% and 12.3%, respectively. In 2023, the conversion rate decreased to 14.2% [10].

Our TKA conversion rate was higher than that reported in the literature. We observed that in one patient, the trochlear component was placed with excessive external rotation, and the rescue procedure was converted to TKA rather than revision, as described in some publications. Of the remaining seven failures, two had concomitant KL II femorotibial OA with established osteophytes prior to PFA implantation. Although the correlation between femorotibial KL II with established osteophytes and the risk of TKA conversion is not quantified, we believe it is an important risk factor, and that correlation should be investigated. Although this does not allow us to draw consistent conclusions, in our PLFIR historical cohort, even a presurgical KL I had a 5 (95% confidence interval: 2.1–12.2) OR of failure when compared to KL 0 [20]. In the PFA series, only two patients had KL II OA with established osteophytes, and both required TKA conversion. If the patient with the implant malposition had been revised and the two cases with femorotibial OA KL II had been excluded, the conversion rate to TKA would have been 16.13%, lower than that reported in the Australian registry for the same year.

The causes of failure in our PFA series follow a pattern similar to that described in the literature, with femorotibial OA progression and persistent pain being the main causes, accounting for 62% and 25%, respectively, while the remaining 13% were due to mechanical problems with the implant [27]. The fact that patients requiring TKA conversion were older and had a more varus alignment might indicate that a borderline indication with incipient medial femorotibial OA could be associated with faster disease progression and thus earlier failure than in patients with normal alignment [21, 28], although our study did not have enough power to either confirm or dismiss association.

The survivorship of the two cohorts at 10 years is similar, with no statistically significant differences. Although a greater risk of failure is observed in the first 5 years for the PFA group, this trend is reversed in the long term, with a decrease in the survivorship of the PLFIR group while PFA survival rates are maintained. It is important to note that four of the eight PFA failures occurred in the first year, mainly due to persistent pain. Given the asymmetry of the two cohorts, with 2.5 times more patients in the PLFIR group, these four cases of failure may be overrepresented, and therefore the PFA survival rates may be underestimated.

Osarumwense et al. reported [23], published in 2017, the outcomes of the first and only case series using the Zimmer Gender Solutions Patellofemoral Joint System. Thirty‐six patients (49 knees) were followed for 40 months (24–58 months). Only two patients (4.1%) required TKA conversion, at 26 and 29 months, both due to persistent pain and mild progression of femorotibial osteoarthritis. Compared to our cohort, three times as many patients required a revision within 58 months, with only one patient requiring a revision after 5 years. According to this data, the first 4 years are crucial in determining the survival rate of PFA.

This study has several potential limitations. The first is due to the design itself, as comparing a current series with a historical cohort introduces inherent biases. Even so, we believe these are minimal, given that the quality of the historical registry was good, the same eligibility criteria were used in the current series, the demographic variables of both groups are comparable, and post‐operative management was the same. However, outcome variables were collected by different senior knee surgeons with intrinsic bias. Indications for the two procedures were comparable as in our centre PLFIR was replaced by PFA following the trend of standard of treatment across Europe. It is worth emphasising that the only variables that were statistically different between PFA and PLFIR were PTA, C‐D index and TA‐GT. Upon review, we found that these differences were minimal and always within the normal range. All radiological measurements were taken by one senior knee surgeon and thus this difference could be justified by bias inherent in intra‐observer variability.

Other possible limitations, for which we do not have a solution, include the differences in the timing of sampling and sample sizes.

## CONCLUSION

PFA (Gender Solutions, Zimmer) demonstrated comparable survival rates to PLFIR in the treatment of IPFOA. Despite a higher initial failure rate and a need for conversion to TKA within the first 5 years, PFA scored significantly better in patellofemoral‐specific tests in the long term, with a potential for greater improvement, particularly in terms of anterior knee pain.

## AUTHOR CONTRIBUTIONS

Jan Martínez‐Lozano: Principal investigator, responsible for data collection and manuscript writing. Lucas Martorell‐de Fortuny: Principal investigator, responsible for study design, data collection and manuscript writing. Lidia Ana Martin‐Domínguez: Conceived the study idea and contributed to data collection. Raul Torres‐Claramunt: Contributed to study design and manuscript writing and served as one of the surgeons. Juan Sanchez‐Soler: Served as one of the principal surgeons and contributed to data collection. Simone Perelli: Collaborated with methodology design and reviewed the manuscript. Pedro Hinarejos: Served as one of the principal surgeons and reviewed the manuscript. Joan Carles Monllau: Conceived the study idea, supervised the study and reviewed the manuscript.

## CONFLICT OF INTEREST STATEMENT

The authors declare no conflicts of interest.

## ETHICS STATEMENT

The study received the institutional review board's approval of our Ethical Committee (2023/14‐TRA‐DEX), signed on 1 August 2014 (acta no. 16/2024). All patients provided written informed consent to participate in this study.

## Supporting information

Supporting information.

## Data Availability

The data sets generated during the current study are available from the corresponding author on reasonable request.
