# Supplementary figures and images for: Patellofemoral arthroplasty provides similar long‐term survival rate and complications with better clinical outcomes compared to facetectomy for the treatment of isolated patellofemoral osteoarthritis
Source: J Exp Orthop. 2025 Jan 10;12(1):e70136. doi: 10.1002/jeo2.70136 (PMC11718545; doi:10.1002/jeo2.70136)

Figure to use in social media


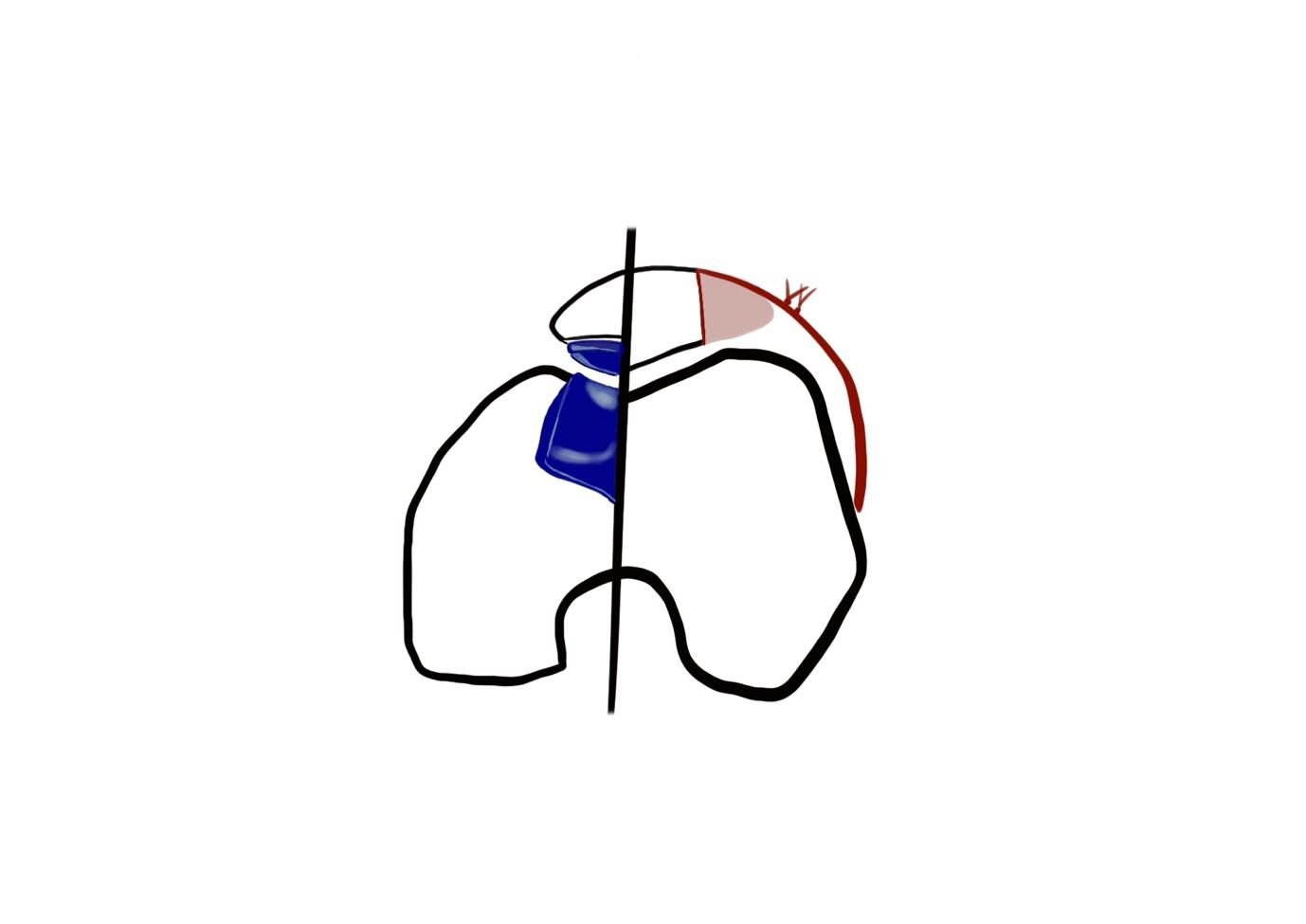

Supplement: Supplementary file 1 — Supporting information. [file JEO2-12-e70136-s001.docx]
